# Exploring the Role of Visual Design in Digital Public Health Safety Education

**DOI:** 10.3390/ijerph18157965

**Published:** 2021-07-28

**Authors:** Minzhe Yi, Defu Bao, Yifan Mo

**Affiliations:** 1 School of Art and Design, Zhejiang Sci-Tech University, Hangzhou 310018, China; myf0805@163.com; 2 Institute of Zhejiang Sci-Tech University-Ouhai, Wenzhou 325000, China

**Keywords:** health education, digital health, visual design, COVID-19

## Abstract

In this research, the positive role of interface visual design in digital safety education was verified taking COVID-19 prevention and control knowledge as the content of public health safety education, where interface emotion (positive, negative, and neutral) and interface layout (waterfall typed and juxtaposition typed) were regarded as independent variables, and readers’ understanding, course evaluation and system usability score were dependent variables. As revealed in the results of a 3 × 2 two-factor experiment in which 252 college students participated: first, different interface emotion can cause significantly different understanding, where negative emotion has the best learning transfer effect; second, due to the difference in interface emotion, participants may give certain courses significantly different evaluation scores, while positive emotional interface contributes to the obviously high scores of three course-evaluation items, “appeal of the lesson”, “enjoyment of the lesson” and “interface quality”; third, significantly different system usability can be caused by different interface layout, where waterfall-type layout enjoys higher appraisal from users; fourth, interface emotion and interface layout have a similar interactive effects in terms of “effort of the lesson” and “interface quality”, where waterfall-type layout is favored in terms of positive emotional interface, and juxtaposition-type layout is more advantageous in terms of negative emotional interface. These results are of vital significance for interface design and safety education. Further, the visual design method for interface emotion and interface layout were analyzed to determine the most suitable design principles so as to improve the effect of digital public health safety education and provide constructive ideas for fighting against COVID-19 at the educational level.

## 1. Introduction

Safety education refers to the way to arrive at the knowledge necessary for safe conduct in daily life or specific activities, through education. Providing employees with safety education is not only a legal obligation, but also an opportunity for a company to ensure the safety of employees and others [1]. There have already been plenty of research results in safety education. For instance, Fransman et al. [2] proved that computer simulation technology can be used in traffic safety education to improve people’s understanding of traffic signs. Turgut et al. [3] studied the methods of providing students aged 10–14 with water safety education on land, and surveyed the impact of different urban environments on children receiving water safety education. Song & Han [1] studied the influence of gender, age, experience and similar factors on teachers receiving online safety education. It was reckoned that only if teachers have deep and accurate understanding of the content of safety education can they play a good demonstrative role and pass on correct safety knowledge to students. Safety education is a broad concept, covering many fields such as traffic safety, life safety, occupational safety, and health safety. Although a lot of valuable experience has already been accumulated, there is still extensive space for research in the subdivisions of safety education.

Digitalization has become an inevitable trend in education industry. Digital public health safety education aims to spread public health knowledge widely to people through digital platforms (such as websites, smart phones and tablets), and its advantage is in guiding readers to learn independently and in easy update of their learning content [1]. Taking digital public health safety education as the research scope, this article focuses on the safety education relevant to prevention and control of COVID-19 which is of great significance for both protecting personal life safety and restoring normal economic and social operations in countries around the world.

In this paper, an analysis will be made, from the perspective of interface visual design, on how to improve the effect of digital health safety education via smartphone in order to better popularize COVID-19 related protection knowledge, because 80% of the information processed by the human brain comes from vision [4]. The essence of education refers to the cognitive process in which people learn unfamiliar knowledge and transform unfamiliar knowledge into acquired knowledge. The influence of emotion and layout factors in the interface on these cognitive process has been confirmed by many studies [5,6,7,8,9].

Emotion is a psychological and physiological status and the result of human thinking after subjection to an external stimulus [10]. Emotional design aims to convey enjoyment, pleasure, trust, satisfaction and other emotions to users through visual elements such as images or colors, aiming to cater to users’ emotional needs [11]. Lockner and Bonnardel [12] stated that in addition to satisfying basic operational functions, visual design should also convey emotions (especially positive emotions) through visual interaction. Based on this, two new research ideas are extended: understanding the process of the generation of emotions, and how to apply interface design to arouse conscious or unconscious emotions. However, in visual design it is not easy to adopt emotional design, because the expression of both positive emotions and negative emotions will be affected by a large number of design details [13].

Layout design aims to present information in a meaningful visual form and expand the user’s visual range [14]. Poor layout may cause confusion in the information architecture and increase the cognitive burden on users, thereby reducing the efficiency of users in operating equipment [8,15]. Ziefle [8] illustrated that a meaningful layout can help users navigate the interface and easily identify information that matches their needs. Among the abundant research on information seeking, many scholars have reported that the interface layout is a significant factor affecting users’ task performance, and plays a significant role in the speed and accuracy of locating specific information [9,16,17]. Therefore, ensuring that the interface layout organizes information in a way that users expect so that relevant content can be easily identified is critical to improving the effectiveness of digital education.

During the pandemic, digital technologies played a role in enabling public-health messaging at scale (e.g., real-time reporting of confirmed cases and deaths using data dashboards). However, relevant research focuses on population surveys, case identification, contact tracing and other fields [18], and there is no guide for the emotional or layout design of the interface. Therefore, this present study aims to discover the best interface visual design scheme by analyzing the influence of different emotional designs and layout designs on people’s knowledge of COVID-19 protection.

## 2. Related Work

To the best of our knowledge, there is scarce research on the use of emotional design and layout design in smartphones to promote health safety education. On the one hand, many studies choose computers (Web or Microsoft PowerPoint) instead of smartphones as the communication medium for public health safety education. With different hardware and interactive modes, computers and smartphones lead to different cognitive loads [19] and decision-making behaviors [20], as well as many other differences. On the other hand, even if there is some research on the design of public health safety education on smart phones, the authors do not explicitly point out that visual design is based on emotional or layout factors, but use such keywords as “user experience”, “attractive” and “aesthetic” to describe their design concept [21,22]. Therefore, this present study explored the interface visual design method with reference to education about Covid-19 protection on smart phones from the perspective of emotion and layout, as a response to the academic gaps in this field.

### 2.1. Emotional Design in Interface Design

It is becoming more and more important to provide appropriate emotions in the practical interface, because emotional design can enhance the trust [23] and attention [24] of users, in addition to improving task performance [25]. According to emotional design principles, the use of attractive visual elements can improve cognitive ability in the learning process, help learners better understand the learning materials, and thus enhance learning outcomes [26]. Lockner and Bonnardel [12] proposed an emotional interface model which illustrated that interface emotion was mainly composed of three closely related specific components: content, interface design and task. Many elements in the interface design (i.e., voice, color, image, etc.) may influence interface emotion. Here, only research on emotional design relevant to colors and facial expressions is expounded for subsequent empirical study.

#### 2.1.1. Conveying of Emotion by Color

As revealed in extensive research on the correlation between interface color and emotion, color plays a vital role in interface design from the aesthetic and pragmatic perspectives. Andersen and Maier [24] studied the relationship between color and attention to explore the best color suitable for a specific search task. Eventually, they found that primary colors could attract more attention from people than secondary colors, where red attracted the most attention and purple and orange got the least attention. In interface design, colors can also resonate with other sensory elements, influencing user emotions together. As demonstrated in the research results of Wu et al. [27], the emotional state of potential online shoppers varies with different music and colors. When fast-paced music is played and red background is shown on an online store, the user feels stronger arousal and pleasure. It is speculated that the result may be affected by cultural background. It may be because red is a color for conveying a “merry mood” in Chinese traditional culture so that red can bring the user greater sense of pleasure. Unlike many researchers concerned with the impact of a single color on interface emotion, Papachristos et al. [28] used a Bayesian Network to produce color combinations that could effectively convey interface emotions. Taking a news website as an example, the tools developed by the researchers can recommend a suitable interface color matching scheme for emotional values such as “consistent”, “reliable”, and “subjective” that could be employed in industry.

Many researchers have also conducted studies on the relationship between interface color and sense of trust, because in an e-commerce environment, users’ consumption behavior depends on their initial sense of trust in unfamiliar webpage interfaces and this sense of trust is often affected by the interface color [29]. In an early piece of research, it was proposed that the dominant tone of an online bank interface should be cold rather than warm. Bright background colors and unbalanced color schemes might intensify users’ sense of distrust [30]. Cyr et al. [31] also confirmed that interface color was an important factor affecting users’ trust and satisfaction with a webpage, and different effects were produced according to different cultural backgrounds. Germans prefer blue, Canadians prefer gray more than Germans and Japanese do, and Japanese do not like webpages with a bright yellow tone. Pelet and Papadopoulou [32] analyzed the influence of e-commerce website color matching on consumers’ emotion, memory and purchase intention and found that negative emotions could enhance memory but reduce purchase intention. Hence, reducing the contrast between background color and foreground color is conducive in increasing consumer’s memory of the commercial information shown in the interface. The more the commercial information is memorized by the consumer, the stronger the willingness to buy.

Although various colors have strong or weak impacts on interface emotion, red and blue seem to have received more attention from researchers than other colors. As pointed out by Mehta and Zhu [25], red gives people the feeling of danger and alertness, while blue is usually associated with emotions such as openness, calmness and tranquility, so that the two colors have different impacts on task performance. Namely, red can enhance detail oriented task performance, while blue can heighten creativity oriented task performance. This conclusion is consistent with people’s general preference for blue over red. In the research of Hawlitschek et al. [29], an analysis was made of the role of red and blue as dominant tones in a game interface. As a result, red was regarded as warm, while blue was cold. Basically, most researchers held that red and blue produced opposite emotions. However, Setyohadi et al. [33] disagreed with this view. They found that red and blue in a mobile learning interface could both bring positive emotions and blue could stimulate the user to produce this positive emotion to the maximum.

#### 2.1.2. Conveying Emotion by Facial Expression

Due to the intrinsic expressiveness of facial expression, a brief glance especially in social situations is enough to quickly acquire the age, gender, social status and other important information [34]. Since Parke [35] tried to make changeable virtual faces on the computer, digital faces have been widely used in electronic games and movies. As revealed in the research of Ekman and Friesen [36], human beings generally have six basic emotions, anger, disgust, fear, happiness, sadness and surprise, conveyed in the verbal and nonverbal social interaction of human beings through facial expressions. Therefore, Ekman and Friesen developed a Facial Action Coding System (FACS) to describe any facial expressions that humans can make as anatomical action units (AUs). This system has become widely used by emotion researchers [37].

How to use facial expressions to convey emotions in interfaces has received widespread attention from researchers. Dyck et al. [38] discovered that the facial expressions created by virtual reality technology could hardly convey the sense of disgust, although they could easily convey the sense of sadness and fear. As pointed out by Danev et al. [39], the mouth is the key element for conveying emotions, but may lead to confused emotional expression. Via the mouth, neutral emotions have the least recognition degree, while anger is easiest to be expressed. García et al. [40] studied the engineering principles for virtual characters to express emotions and tested the ability of healthy people to recognize virtual facial expressions. The result showed that the basic emotions conveyed by virtual facial expressions had an overall accuracy of 88.25%, where the recognition accuracy of disgust was the highest. In the researchers’ opinion, the time has come to use virtual faces to completely convey interface emotions, especially for high-intensity emotional expressions. Mudrick et al. [41] systematically observed the impact of a virtual tutor’s facial expressions on students’ learning performance, and verified that this impact indeed existed as students would evaluate themselves based on the tutor’s facial expressions. If the virtual tutor made neutral expressions, students’ confusion would be intensified.

#### 2.1.3. Research on the Influence of Emotional Types on Learning Outcomes

Although there have already been some design guidelines for arousing user emotions through interface design, it is still not decided which kind of emotional design can create the best user experience. Many researchers have maintained that in the learning process, creating positive emotion was more conducive to improving learning efficiency than negative emotion [5,11,42]. Heidig et al. [43] explored the visual design features that lead to positive and negative emotions in online education, finding that positive emotion design can promote readers’ retention and comprehension, whereas negative emotional states did not have the expected positive effect, but instead hindered learning. This point of view was advocated by Norman, who thought that the designer must induce positive emotion such as trust in order to provide good user experience, while negative emotion such as anxiety meant negative user experience [44]. On the other hand, some researchers believed that negative emotions also had their value. Kumar et al. [45] considered that so-called negative design had not been fully studied because it sounded absurd and violated design principles, but it could be asserted that an interface with negative design did not imply that it had negative usability. Moridis and Economides [6] affirmed the value of negative emotions, on the grounds that negative emotions were conducive to improving readers’ concentration in learning.

### 2.2. Layout in Interface Design

Layout design aims to plan the organization of interface information, in order to help users browse content efficiently, reduce users’ tasks and cognitive load, and try to solve the problem of disorientation faced by many users [15]. Although the research on interface layout spans many fields, such as online education [17,46,47], e-commerce [48,49] and Man-Machine systems [50,51], its core idea is to optimize the processing process when users receive information, and to improve task performance. A key element in layout design is that the layout needs to meet the information search strategies of different groups. Young people and old people are two typical groups, and their information search strategies are different. For example, young people will adopt different search strategies according to different tasks, but old people are accustomed to using top-down search strategies no matter what tasks they face [17,47]. Therefore, the interface layout for the elderly should be as concise as possible to reduce the up-and-down scrolling of pages and ensure consistency of information organization when users switch between different interfaces [47].

In the field of e-commerce, because consumers need to choose products that meet their needs from a large number of substitutes, interface layout that presents commodity information determines whether consumers can order their desired goods. Sulikowski and Zdziebko [48] found that vertical layout is more attractive than horizontal layout in commodity recommendation systems, and the least attractive positions in the interface are at the bottom of the interface (vertical layout) and the right edge of the interface (horizontal layout). Barbier et al. [46] stated that users tend to pay more attention to the visual elements in the middle of the interface. They stressed that the key information should be arranged on the interface where the human eye sees it first in the natural state, which can force the user to pay direct attention to the content. In addition, increasing brightness and contrast can further improve the readability of the interface. Another study has confirmed that interface layout can significantly affect consumers’ shopping time, and there is an interaction between the interface layout and educational background. People with lower education level reject an interface that provides too many choices, because it is not in line with their usual offline consumption habits; on the contrary, people with higher education preferred an interface layout offering rich options [49].

Consoles designed for an operating space (e.g., aerospace manned cockpit, car cab) in a closed environment and their layout design have also received attention because the users of this space are often professionals rather than ordinary people, who need to make rapid and accurate judgments based on the interface information prompts. Therefore, a reasonable layout should conform to information organization rules and visual search strategies, so as to improve the recognition ability and work efficiency of operators, while an improperly designed layout may lead to incorrect operation and occupational diseases. Yan et al. [50] believe that the layout design should be consistent with the user’s past experience, which can reduce the user’s learning time and error probability. Li et al. [51] summarized the layout principles of the man–machine interaction interface in a cockpit from the perspective of cognitive psychology. For example, relevant information should be put together according to the proximity principle or marked with the same color according to the similarity principle. They also argue that the interface layout problem is essentially an optimal combination problem, and designers need to select an integrated layout scheme that best meets the task requirements from different combinations.

## 3. Research Questions and Hypotheses

This research was inspired by the work of Mayer and Estrella [26], in which emotional design was applied to multi-media courses to explain “how common cold virus attacks the body” to students. As found by the researchers, emotional design contributes to improving students’ understanding of medical knowledge and motivates them to make more effort in learning. However, Mayer and Estrella did not classify emotional design. It is unknown whether different emotional design can have a different impact on the cognition process.

In view of the controversial influence of emotion types on learning outcomes, we tend to support the view that different emotion types have their own advantages. As pointed out in the research of Moridis and Economides [6], positive emotion may distract learning attention while promoting the cognition process; on the contrary, assuming that negative emotions (stress, anger, etc.) hinder the cognition process, students’ concentration in learning may be enhanced by these emotions. Kumar et al. [45] indicated that the learning achievement of students was improved by applying both positive and negative emotional design to the interface. The results of research by Chung and Cheon [52] showed that, although positive emotions can expand people’s attention span, negative emotion design facilitates the allocation of more cognitive resources.

To illustrate our point in the context of digital health safety education and explore the role that interface layout plays, we decided to apply the theoretical framework to emotional design principles [26,53] and a layout design model for a human–machine interaction interface [51,54], and we designed three interface emotions (positive, negative, and neutral) and two interface layouts (Juxtaposition and Waterfall) to help people understand the relationship between interface emotion and interface layout, and the impact of the two on learning outcomes. Based on the literature and previous findings, these research questions were formulated.

- How do different interface emotions affect participants’ learning outcomes?
- Which interface layout is best for improving participants’ learning outcomes?
- What is the interaction between interface emotion and interface layout?

The learning outcomes measured in this study are understanding (cognitive assessment), course evaluation, and system usability (emotional assessment). Since emotion and cognition are closely associated [44], it was essential to have both measurements. The hypotheses were selected as null hypotheses, because we have not found any studies that analyze the impact of interface emotions and interface layout on learning about COVID-19 protection via smartphones. The hypotheses for this study are:

**Hypothesis** **1.** *Learning about COVID-19 protection on smart phones will not lead to significant differences in participants’ understanding, course evaluation and system usability due to different interface emotions.*

**Hypothesis** **2.** *Learning about COVID-19 protection on smart phones will not lead to significant differences in participants’ understanding, course evaluation and system usability due to different interface layouts.*

**Hypothesis** **3.** *There is no significant interaction between interface emotion and interface layout.*

## 4. Materials and Methods

With the advent of the mobile internet wave, people have become accustomed to obtaining information on mobile devices. Hence, it is expected that the best way to provide public health safety education is via smartphones and the understanding of the role of interface design. In this research, a 3 (interface emotions) × 2 (interface layouts) two-factor experimental design was adopted and 6 interfaces were designed, where, the dependent variables included participants’ understanding, course evaluation and system usability score. This research was not only to simply discuss the impact of emotional design on dependent variables, but also to classify interface emotions and further analyze the specific advantages of different interface emotions in health safety education. Here, interface emotions were divided into positive, negative, and neutral emotions. This classification criterion was also used in previous researches on emotional design [6,42,45]. Moreover, interface layout was used as another independent variable, because studies have shown that interface layout affected users’ cognitive abilities, such as in automotive multimedia systems [15]. The design strategies for interface emotions and interface layouts will be introduced in Section 4.2.2 in detail.

### 4.1. Participants

In this research, 252 college students (119 men and 133 women) were recruited as participants from local universities in southern China via a purposive sampling method. Most of them were sophomores, and all of them are volunteers without pay. The reason for this sampling is that the university is a highly populated place and most university students in China are required to live on campus so that they urgently need to receive public health safety education. All participants are randomly assigned into six groups, each with 42 people, and each group of participants only tested one of the six interfaces to avoid the influence of experience on the test results. All participants have no medical professional background, and the biological knowledge of each group of participants has no statistically significant difference. The biological knowledge questionnaire consists of seven items. The 5-point Likert scale score was used, ranging from very low (1) to very high (5). The sum of all items scores was the subject’s biological knowledge score: (1) I participated in a biology related research group; (2) In high school, biology was my favorite subject; (3) I would watch science and education channels in my spare time; (4) I can name most of the cell’s organelles from memory; (5) Sometimes, I would search the internet for knowledge relevant to biology; (6) I have (or once had) a microscope; (7) I took advanced biology classes in high school. Mayer [53] used a similar knowledge questionnaire to measure the level of prior knowledge of participants without the need for asking specific questions about the content of the experiment.

### 4.2. Apparatus and Prototype

The study used a “MockingBot” for interactive prototyping (a popular interface prototyping tool in China), and “Photoshop” and “Illustrator” for image processing. The interactive prototype was developed for the iPhone 11 Pro Max, with a 6.5-inch screen, 1242 × 2688 resolution, and 458 pixel density. The simulation interface developed through “MockingBot” may run on a mobile phone, and so the participants operated on real phones rather than computers.

#### 4.2.1. The Content of Digital Public Health Safety Education

COVID-19 related protection knowledge was taken as the content of public health safety education. As COVID-19 has had a huge impact on human society, including China, it is particularly important to master scientific knowledge of virus defense to prevent the spread of the epidemic and protect personal health. The “Coronavirus disease (COVID-19) advice for the public” [55] shown on the Chinese official website of the World Health Organization (WHO) was taken as the learning content, as Chinese was the mother tongue of the participants (accessed on 16 March 2021). With the deepening of research on COVID-19 by medical institutions in various countries, COVID-19 related protection knowledge may be updated. However, the focus of this research was to analyze interface design methods to promote understanding of professional medical knowledge, with an expectation of influencing the popularization of new COVID-19 related protection knowledge delivery in the future.

#### 4.2.2. Interface Prototype Design and Manipulation Check

Three illustrators and two interface designers were invited to complete the interface prototype design together. First, the illustrators created comics relevant to COVID-19 protection knowledge. Then, the interface designers had the comics produced into six types of interface for testing. Instead of real faces, a simplified comic style was adopted in the interface. This not only makes it easier to control facial expressions, but also avoids misinformation [39].

In this research, interface emotion was one of the independent variables. According to emotional design principles [26], human-like characteristics and appealing colors can promote the transfer of interface emotions. In view of this, we chose cartoon characters’ facial expressions and colors to convey interface emotions. A large number of researchers pay extensive attention to triggering learners’ corresponding emotions by manipulating facial expressions and colors. For example, they have explored the influence of virtual teachers’ facial expressions on learning performance [41], or analyzed what colors can trigger positive emotions in the process of mobile learning [33]. Young et al. [56] confirmed that color can enhance the emotional transmission effect of facial expressions.

**Figure 1:**
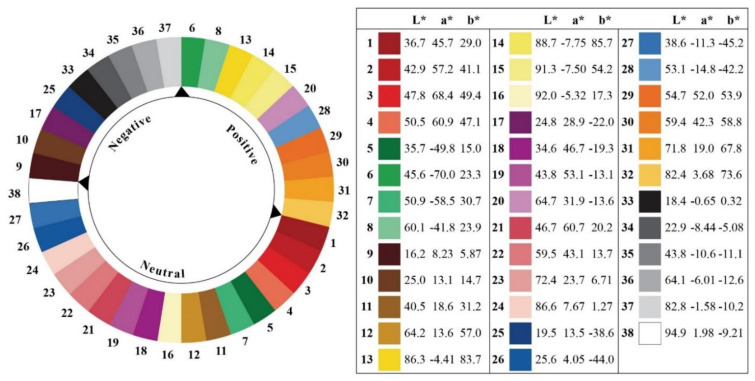
The color matching scheme used in this research. Colors are shown with their corresponding L*a*b* D50 coordinates (CIE 1931; 2 degree Observer).

**Figure 2:**
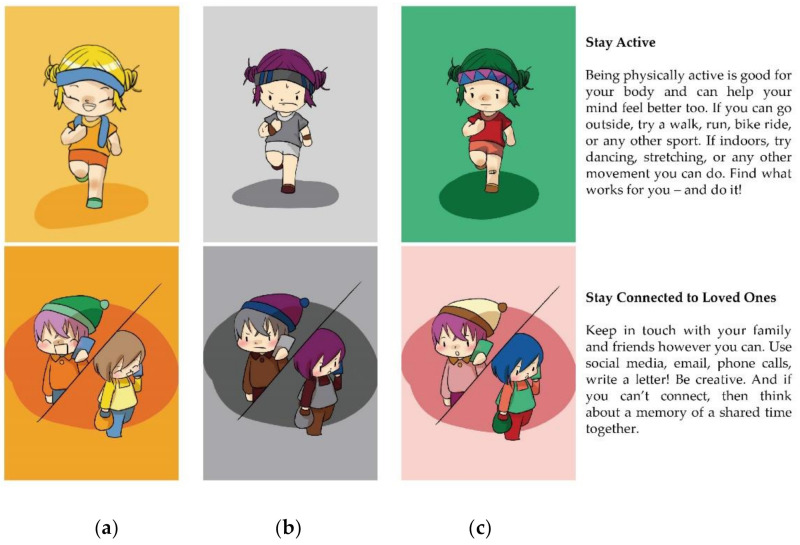
Examples of comics used for propagating the COVID-19 related protection knowledge (the text has been translated into English, and all texts are the same regardless of the emotional design): (**a**) Comics based on positive emotion design; (**b**) Comics based on negative emotion design; (**c**) Comics based on neutral emotion design.

In drawing facial expressions, illustrators adopted the previous methodology used by Danev et al. [39] who gave suggestions on how to use simplified, comic-style faces to convey emotions. After finishing the black-and-white version of the comics, we used a validated color scheme [57] to color the black-and-white version. This color scheme was produced by researchers at the University of Manchester to establish a reliable and validated relationship between color and emotion. With the participation of 204 healthy volunteers, 38 colors in the color wheel were divided into three categories according to different emotional attributes, positive, negative and neutral, and the research result was of high reproducibility (Figure 1). The colored cartoon is shown in Figure 2.

**Figure 3:**
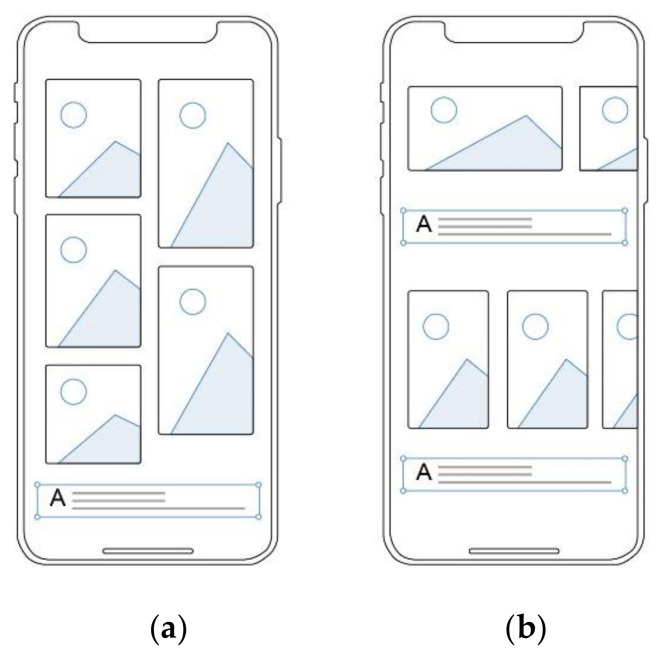
Spatial analysis diagram of interface layouts. (**a**) Waterfall-type; (**b**) Juxtaposition-type.

Interface layout was another independent variable. As we have introduced in related work, interface layout can affect people’s cognitive process, which has been confirmed by multiple research fields [47,49,51]. Considering that all of our subjects were Chinese, the interface layout should conform to the user’s habits and experience from the perspective of cognitive psychology [51], so this study analyzed 26 common interface layouts of digital education Apps in China rather than in overseas markets as the source of inspiration for layout design. In the process of layout design, we also followed the layout principles based on cognitive psychology [51,54]. For example, in order for the information to be presented clearly, the relevant information should be put together according to the proximity principle. Finally, we created two types of interface layout for the empirical study and named them Waterfall Type and Juxtaposition Type (Figure 3). Among them, the information modules in waterfall-type layout were in uneven multi-column distribution so that the user could select learning content by sliding the phone screen up and down, resulting in a high space utilization rate of the interface, while the information modules in juxtaposition-type layout were arranged repeatedly up and down or left and right so that the user could select learning content by sliding the screen up and down or left and right.

**Figure 4:**
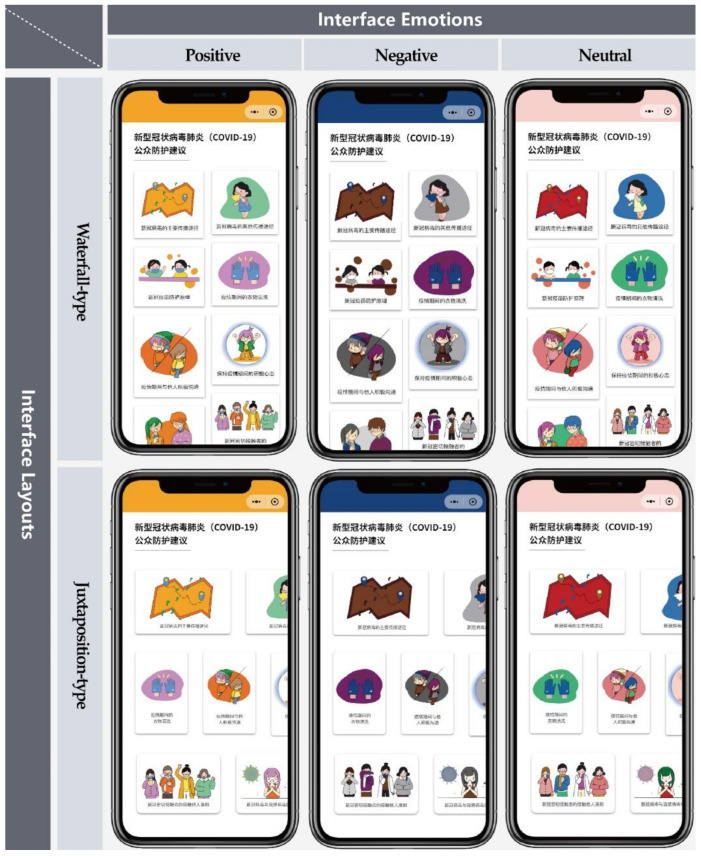
Homepages of the 6 interfaces designed in this research (in Chinese). Participants can click on each information module to learn more about virus protection knowledge.

The six types of interface designed jointly by illustrators and interface designers are provided in Figure 4, in order to know about the impact of different interface designs on people’s learning of COVID-19 protection knowledge and seek the optimal interface design scheme. Users’ evaluation and response are usually based on overall impression rather than specific elements of the interface [23]. Therefore, we performed a manipulation check (i.e., emotion recognition test) prior to the formal test to confirm that different interface designs can convey the three emotions we want in the overall impression.

A manipulation check was conducted at a subway station near the university. Respectively, 15 college student passengers were randomly invited for each type of interface and asked to select an emotional vocabulary suitable for the interface or select “none of the above vocabulary is correct”. There are three stages in choosing the vocabulary. First of all, we selected 34 words from the related research on Chinese emotional words [58,59] to constitute the initial version of the vocabulary, including 11 positive words, 10 negative words and 9 neutral words, all of which are two-word words. Then, 35 college students assessed the valence and arousal of all the items of vocabulary using the Likert seven-point scale. The results of variance analysis showed that there were significant differences between the three kinds of emotional words in valence (*p* < 0.001); there was no significant difference in arousal between positive and negative words (*p* > 0.05), but there was a significant difference between these and neutral words (*p* < 0.001). Finally, in order to examine the typicality of these emotional words, we conducted a questionnaire survey among 98 college students, asking them to evaluate whether they had had the emotional experience described by these 34 words in the past month. The final version of the vocabulary includes four positive words, three negative words, and three neutral words, because more than 90% of people reported having experienced the emotions described by these words.

Manipulation check results are shown in Table 1. All the words have been translated into English, the recognition rate of neutral emotional interface with waterfall-type layout is 86.7% and that of neutral emotional interface with juxtaposition-type layout is 80%, while those of the remaining four types of interface design are 100%.

### 4.3. Experimental Procedure

**Figure 5:**
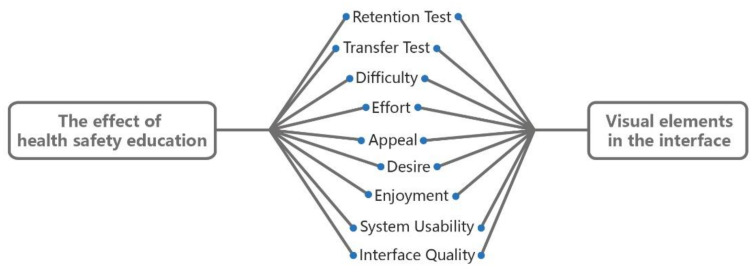
The evaluation framework associated with the educational effect in this research.

252 participants were randomly divided into six groups, each with 42 participants. In order to eliminate unnecessary interference factors, the experimental site was located in a quiet classroom. The experimental procedures of each group were the same, including three stages. In the first stage, a research assistant introduced the interface operation method and experimental content to each group of participants and answered their questions. In the second stage, participants were asked to read all the COVID-19 protection knowledge education content in the interface within 15 min. Each group was provided with one set of iPhone 11 Pro Max with a safety education prototype system and thus participants needed to finish the learning task by using this smartphone in turn. In the third stage, participants needed to take an understanding test after completing the health safety education, and to fill out the course evaluation questionnaire and Post-Study System Usability Questionnaire (PSSUQ). Ethical principles were adhered to in conducting research with human subjects. The evaluation framework adopted in this study is as shown in Figure 5.

The understanding test consists of retention and transfer tests. The retention test only has one question: “Based on the reading content, please describe how to wash and dry clothes, towels and bedding when there is a suspected or confirmed COVID-19 patient in your home”. In this test, a correct answer of every knowledge point as shown below is scored 1 point, the maximum score accumulated is 11 points and the minimum one is 0 point, regardless of specific wording: (1) Wash the patient’s clothes, towels and bedding separately; (2) If possible, wear gloves before handling the articles; (3) Never carry these dirty supplies next to your body; (4) These supplies should be placed in clearly marked non-leaking containers (such as bags or buckets); (5) Before putting these supplies into the special containers, use a flat and hard instrument to scrape the solid excrement (such as feces or vomit) from these supplies into the patient’s close-stool; (6) If no close-stool is provided in the patient’s room, scrape the excrement into a bucket with the lid, and then take it to toilet for disposal; (7) The articles should be washed in the washing machine with hot water (60–90 °C) and detergent; (8) Have the articles soaked in a large bucket with hot soapy water and then stirred with a stick, but do not tap it to splash; (9) If there is no hot water, soak in 0.05% chlorine bleach solution for about 30 min; (10) Finally, rinse with clean water, and then dry in the sun; (11) Do not forget to wash your hands, finally. As a result, two raters scored the retention test, with an inter-rater reliability of r = 0.83, and disagreements were resolved by consensus.

According to COVID-19 Protection Knowledge published by the WHO in its official website [55] and the suggestions on transfer tests in previous relevant studies [26,60,61], we developed the initial version of the questionnaire for the transfer test. Subsequently, the questionnaire items were evaluated by the Delphi method based on seven experts, four of whom were clinicians, and the other three health education and health promotion experts. They were from the school clinic and hospital. All had received training in COVID-19 epidemic prevention and control, and had a positive attitude to the study. After two-round expert consultation (with a 100% recovery of responses), we revised the content and sentences of the initial version of the questionnaire based on expert comments, which were unanimously endorsed by the experts. The final version of the questionnaire includes five questions: (1) Assume that you once were in the same indoor space (such as an office) with a COVID-19 patient, but you were not infected with the virus. What are the possible reasons? (2) Your friend becomes very anxious because of the impact of COVID-19 on life. What advice do you think can help him? (3) What should you do if you suspect that you have been infected with COVID-19? (4) If you are vaccinated against COVID-19 in the future, what impact will it have on yourself and the community? (5) Why is COVID-19 similar to seasonal flu in disease symptoms but more dangerous than the latter? In this test, each question has multiple choices. The scoring is an accumulation of the scores of the five questions, while the score of each question depends on the number of correct answers given by the participants and each correct answer is scored 1 point. The maximum score accumulated is 27 points and the minimum one is 0 point, regardless of specific wording. For example, among the “possible reasons” in the first question, there are five acceptable answers, namely: (1) The COVID-19 virus was blocked by medical masks; (2) The indoor space was well ventilated; (3) You maintained a social distance with COVID-19 patient; (4) You didn’t touch desks, door handles, handrails and other objects contaminated by the virus; (5) You didn’t stay with the COVID-19 patient in the same space for a long time. Two raters scored the transfer test, with an inter-rater reliability of r = 0.89, and disagreements were resolved by consensus.

The course evaluation concerns the effects of emotional design on participants’ ratings of affect, effort, or difficulty. Sourced from the research of Mayer and Estrella [26], the course evaluation questionnaire investigates the impact of online course learning on emotional design and consists of five items: (1) “Please rate how difficult this lesson was for you.” on a scale from 1 (“very easy”) to 5 (“very difficult”); (2) “Please rate how much effort you exerted in learning this lesson.” on a scale from 1 (“very low”) to 5 (“very high”); (3) “Please rate how appealing this lesson was for you.” on a scale from 1 (“very appealing”) to 5 (“very unappealing”); (4) “I would like to learn from more lessons like this.” on a scale from 1 (“strongly agree”) to 5 (“strongly disagree”); (5) “I enjoyed learning from this lesson.” on a scale from 1 (“strongly agree”) to 5 (“strongly disagree”).

In this research, a Post-Study System Usability Questionnaire (PSSUQ) was used to evaluate the usability of the interface design and measure users’ perceived satisfaction with the system or product after ending the research. PSSUQ has good validity [62]. We adopted Version 3, which is a 16-item standardized questionnaire, which starts with 1 (strongly agree) and ends with 7 (strongly disagree). User can also mark the prompts as N/A (not applicable). The lower the score, the better the performance and satisfaction.

## 5. Results

In this research, a 3 (interface emotion) × 2 (interface layout) two-factor experimental design was adopted, taking participants’ understanding, course evaluation and system usability score as the dependent variables. As the dependent variables were approximately normally distributed and passed the test for homogeneity of variances, we utilized the Two-Way ANOVA for analyzing relevant experimental data with IBM SPSS (version 24). For significantly different factors, a post hoc test was conducted.

### 5.1. Understanding

Retention and transfer tests provide an overall measurement criterion for understanding, because all items require participants to generate explanations corresponding to level 2 (“understand”) in Bloom’s taxonomy of instructional objectives [63]. The main purpose of the two tests is to determine whether different interface design has significantly different impact on readers’ understanding. In the retention test, participants were required to answer the question about how to wash and dry clothes, towels and bedding when there is a suspected or confirmed COVID-19 patient in the home. In the transfer test, there were five questions in total and participants were required to use known information to explain a problem in a new situation, for instance why a person once staying in the same indoor space as a COVID-19 patient has not been infected with the virus.

#### 5.1.1. Retention Test

Retention is a process by which people acquire knowledge and experience, and the retention quality directly affects the effectiveness of learning. In this test, whether a different interface design had a different impact on improving reader’ retention or not was measured by calculating participants’ number of correct retentions. The higher the score, the better the retention quality of the reader. As revealed in the following Table 2, different interface emotion has no significant different impact on retention (F = 1.215, *p* = 0.298 > 0.05), different interface layout has no significant different impact on retention (F = 0.444, *p* = 0.506 > 0.05), and interface emotion and interface layout are not significantly interactive in terms of retention (F = 1.363, *p* = 0.258 > 0.05).

#### 5.1.2. Transfer Test

Learning transfer represents the ability of readers to construct new knowledge based on existing knowledge. Promoting learning transfer is an important task of teaching activities. The higher the score on the transfer test, the stronger the participants’ ability to master knowledge and apply the learnt knowledge to a new scenario. As can be seen from Table 3, this test score is significantly different (F = 14.264, *p* = 0.000 < 0.05) in terms of different interface emotion, which indicates that interface emotion affects the learning transfer ability of participants; while, in terms of different interface layout, the test score shows no significant difference (F = 3.420, *p* = 0.066 > 0.05), and the interaction between interface emotion and layout in the transfer test scores is not significant (F = 0.974, *p* = 0.379 > 0.05). The result of the post hoc test shows that the transfer test score for the negative emotional interface (M = 20.98, Sd = 2.862) is the highest and is obviously better than the positive emotional interface (M = 19.05, Sd = 2.933) and neutral emotional interface (M = 18.42, Sd = 3.863). This reveals that a negative emotional interface is most helpful for readers to master existing knowledge and apply it to new scenario.

### 5.2. Course Evaluation

#### 5.2.1. Difficulty of the Lesson

According to cognitive load theory, if a reader experiences difficulty in learning, it indicates that the reader bears high cognitive load and needs to make more mental effort to acquire the learning content. This difficulty for participants can be affected by the structural complexity of learning materials, the degree of association between elements, and the organization of teaching materials. The higher the score, the stronger the participants’ feeling of difficulty. The results of descriptive statistics and two-factor analysis are shown in the following Table 4. As revealed, neither different interface emotion (F = 1.617, *p* = 0.201 > 0.05) nor interface layout (F = 2.116, *p* = 0.147 > 0.05) may lead to significant difference in participants’ feeling of difficulty, and the two factors have no significant interaction in terms of the feeling of difficulty (F = 2.634, *p* = 0.074 > 0.05).

#### 5.2.2. Effort of the Lesson

Readers’ effort made in learning is closely related to the learning effectiveness. Score is in positive proportion to the mental energy or attentional resources that reader invests in the learning task. As can be seen from Table 5, different interface emotion has no significant impact on the level of effort made by the participants (F = 2.796, *p* = 0.063 > 0.05), and different interface layout also has no significant impact on the effort level of participants (F = 0.667, *p* = 0.415 > 0.05), while the two factors have significant interaction (F = 14.895, *p* = 0.000 < 0.05). This indicates that the combination of the two factors can affect the level of effort made by the participants.

**Figure 6:**
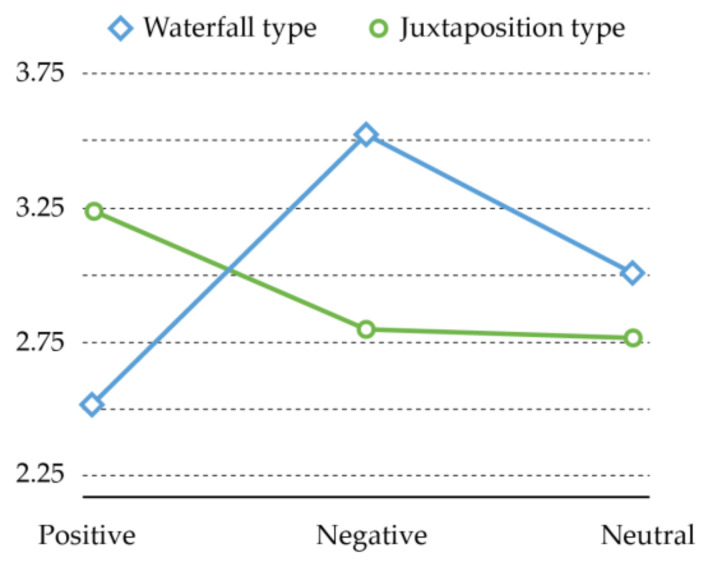
Interaction diagram regarding effort.

The interaction between interface emotion and interface layout can be explained based on Figure 6. As revealed, with positive emotional interface, participants put less effort into waterfall-type layout (M = 2.55, Sd = 0.772) than juxtaposition-type layout (M = 3.24, Sd = 0.850); with negative emotional interface, the latter layout (M = 2.81, Sd = 0.707) is more advantageous, while in the former layout (M = 3.52, Sd = 0.943) participants need to make more mental effort. The advantages of juxtaposition-type layout (M = 2.79, Sd = 0.813) are also reflected in the neutral emotional interface, while in waterfall-type layout (M = 3.02, Sd = 0.975), participants need to make more effort on enhancing retention and thinking ability in learning about COVID-19 related protection.

#### 5.2.3. Appeal of the Lesson

Appeal can stimulate readers to learn appropriately for a certain objective and maintain their learning behavior. Good appeal can make readers eager to learn with initiative, actively and carefully. As shown in Table 6, the lower the score, the stronger the appeal of the lesson. Different interface emotion can lead to significant difference in the appeal (F = 14.355, *p* = 0.000 < 0.05), while different interface layout cannot (F = 1.077, *p* = 0.300 > 0.05). The interaction between the two factors is not significant in terms of the appeal (F = 2.568, *p* = 0.079 > 0.05). The result of the post hoc test demonstrated that the appeal of positive emotional interface (M = 1.76, Sd = 0.786) is higher than that of neutral emotional interface (M = 2.38, Sd = 0.805). Hence, in contrast, negative emotional interface is conducive to readers’ retention and knowledge application, while positive emotional interface has higher appeal to readers.

#### 5.2.4. Desire for Similar Lessons

Desire reflects the motivation and positivity of readers, and can prompt readers to change from passive learning to autonomous learning. The lower the score, the stronger the reader’s interest and curiosity in the learning content. As demonstrated in Table 7, both different interface emotion (F = 0.248, *p* = 0.781 > 0.05) and different interface layout (F = 0.282, *p* = 0.596 > 0.05) have no significantly different impact on the desire to learn. There is no significant interaction between the two factors in terms of desire (F = 0.109, *p* = 0.896 > 0.05).

#### 5.2.5. Enjoyment of the Lesson

Enjoyment of the lesson can reduce the boredom of readers in learning, relieve learning pressure, improve learning satisfaction, and obtain a better learning effect. As presented in Table 8, the enjoyment of the lesson is negatively proportional to the score. Different interface emotion has a significantly different impact on the enjoyment of the lesson (F = 10.009, *p* = 0.000 < 0.05), while different interface layout has not (F = 0.506, *p* = 0.478 > 0.05). In terms of the enjoyment of the lesson, the interaction between the two factors is not significant (F = 2.046, *p* = 0.131 > 0.05). In the post hoc test, positive emotional interface (M = 1.85, Sd = 0.685) brings the strongest enjoyment to participants, followed by negative emotional interface (M = 2.25, Sd = 0.742) and neutral emotional interface (M = 2.29, Sd = 0.704). Positive emotional interface has higher appeal to readers, provides a pleasant and relaxed scene for readers to learn in, and can help readers obtain a greater learning experience and insights.

### 5.3. System Usability

The system usability of the interface was obtained by using PSSUQ. This questionnaire has 16 questions which were grouped into three sub-scales, namely system usability (questions 1 to 6), information quality (questions 7 to 12) and interface quality (questions 13 to 16). Lewis [64] pointed out that focusing on the scores of sub-scales made more sense, as total score of the PSSUQ had limited value. After finishing the understanding test, participants were asked to fill in the PSSUQ. Next, a statistical analysis was made on the scores of two sub-scales (system usability and interface quality) closely relevant to this research.

#### 5.3.1. System Usability

As an important part of user experience, system usability represents the ability of a system to provide universally available functions for users with different physiological characteristics, knowledge backgrounds and skills. It is an index for evaluating the effectiveness of an information system and a key factor that affects the user acceptance of the system. As indicated in Table 9, different interface emotion has no significantly different impact on system usability (F = 1.726, *p* = 0.180 > 0.05), but different interface layout has (F = 39.638, *p* = 0.000 < 0.05), where, the system usability of waterfall-type layout (M = 2.28, Sd = 0.633) is significantly superior to that of juxtaposition-type layout (M = 2.77, Sd = 0.593). The interaction between interface emotion and interface layout is not significant (F = 2.132, *p* = 0.121 > 0.05) with respect to the system usability.

#### 5.3.2. Interface Quality

Interface quality directly affects the efficiency of human–computer interaction and the performance of the system. A high-quality interface has better visual and use effects, so that users can operate the interface more proficiently and even become dependent on the system during use. According to the following Table 10, different interface emotion has significantly different impact on interface quality (F = 14.563, *p* = 0.000 < 0.05), but different interface layout has not (F = 1.935, *p* = 0.166 > 0.05). The two factors are significantly interactive (F = 23.211, *p* = 0.000 < 0.05) in terms of the interface quality, which means that a combination of the two factors can affect the reader’s scoring for interface quality. By post hoc test, it is affirmed that positive emotional interface (M = 2.01, Sd = 0.508) has the best interface quality, followed by negative emotional interface (M = 2.43, Sd = 0.666).

**Figure 7:**
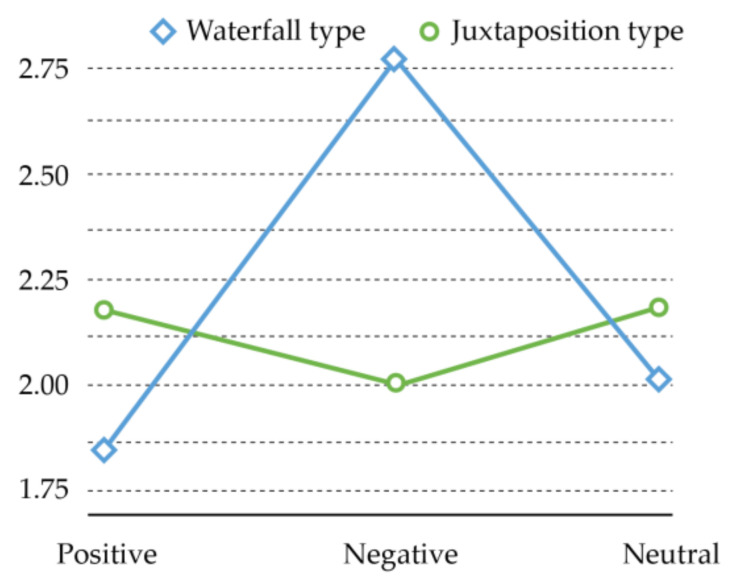
The interaction diagram regarding interface quality.

The following Figure 7 demonstrates the interaction between interface emotion and interface layout. In case of positive emotional interface, waterfall-type layout (M = 1.86, Sd = 0.451) has better interface quality than juxtaposition-type layout (M = 2.17, Sd = 0.518); similarly in neutral emotional interface, waterfall-type layout (M = 2.05, Sd = 0.537) has better interface quality than juxtaposition-type layout (M = 2.18, Sd = 0.465); while in case of negative emotional interface, the interface quality of the latter layout (M = 2.07, Sd = 0.565) is better than that of the former layout (M = 2.78, Sd = 0.564).

## 6. Discussion

In this research, the impact of three interface emotions (positive, negative, and neutral) and two interface layouts (juxtaposition and waterfall types) on participants’ understanding, course evaluation, and system usability under different combinations and the interaction between interface emotion and interface layout were explored. The results show that visual design indeed has impact on the effectiveness of learning about COVID-19 protection.

The first research question was: how do different interface emotions affect participants’ learning outcomes? As found, different interface emotions do cause significant differences in transfer test scores. Negative emotional interface (M = 20.98, Sd = 2.862) scores the highest and is obviously better than positive emotional interface (M = 19.05, Sd = 2.933) and neutral emotional interface (M = 18.42, Sd = 3.863). Our conclusion supports the previous report [6,45], i.e., negative emotions can make the reader’s cognitive process more systematic, while positive emotions can lead to a simplification of the cognitive process and reduce the reader’s learning ability. In memory-based learning, negative affective design has more advantages than positive affective design [32], because negative emotion facilitates the allocation of more cognitive resources [52]. Another reason we think negative emotion performed better in the transfer test is due to the learning content. COVID-19 protection knowledge belongs to professional medical knowledge so that participants often learn about it solemnly rather than for enjoyment, so negative emotional interface caters to this learning mentality. The conclusions of this study on transfer testing are in contradiction to those of Heidig et al. [43] and Park et al. [42], who stated that positive emotion fosters more complex learning goals such as comprehension and transfer, whereas negative emotional states did not have the expected positive effect but instead hindered learning. This may be because our study does not cover the intensity of the emotion, since strong negative emotion may still adversely affect people’s cognition and damage the effect of learning transfer [5,11]. It was not found that different interface emotion affects participants’ retention of COVID-19 protection knowledge. The longer the reader pays attention to the text information, the better the retention effect will be achieved [42], but all participants were asked to complete the learning within 15 min which might be hasty for a small number of participants, so that the role of emotional design in retention was affected.

Among the five questions about course evaluation, positive emotion interface was significantly superior for “appeal of the lesson” and “enjoyment of the lesson”. The appeal of positive emotional interface (M = 1.76, Sd = 0.786) is higher than that of neutral emotional interface (M = 2.38, Sd = 0.805). Similarly, positive emotional interface (M = 1.85, Sd = 0.685) brings the strongest enjoyment to participants, followed by negative emotional interface (M = 2.25, Sd = 0.742) and neutral emotional interface (M = 2.29, Sd = 0.704). This implies that the advantage of negative emotion interface in learning transfer has not been extended to course evaluation. In other words, although negative emotional interface can promote the learning transfer of participants, participants still prefer positive emotional interface. A reasonable explanation for this result is that positive emotion can not only promote individuals to integrate into the environment and participate in activities, but also makes people feel happy and stimulates people’s psychology in playing and exploring [11], which makes the educational content designed with positive emotion have stronger appeal to learners and makes them experience higher enjoyment of the lesson. We find that positive emotional design has certain advantages in subjective evaluation, but this is different from the views of some scholars [42,45], who believe that no matter what emotional design is adopted, there will be no significant difference in subjective evaluation. One probable reason for these inconsistent results lies in the operationalization of the design factor. In addition, different questionnaires for subjective evaluation also lead to different experimental conclusions. In conclusion, Hypothesis 1 is rejected because there are significant differences in understanding, course evaluation, and system usability between different interface emotions.

The second research question is: which interface layout is best for improving learning outcomes? In this research, two interface layouts (waterfall type and juxtaposition type) were adopted, but no significant differences in the understanding and course evaluation of participants caused by different interface layout were found. This might be because the participants were only asked to do one ask in the experiment, namely learning about protection knowledge regarding COVID-19, so that they had sufficient energy to do the task and their understanding was less affected by the difference in interface layout. However, when users do multiple tasks at the same time, the impact of different interface layouts on their understanding will increase due to the fact that their limited energy is decentralized. For example, when a driver operates an on-board music player while driving a car, a different player interface layout may lead to significantly different driving performance and further affect driving safety [15].

Although different interface layouts cannot pose a significantly different impact on the understanding and course evaluation of participants, waterfall-type interface layout (M = 2.28, Sd = 0.633) has significantly superior system usability to juxtaposition-type (M = 2.77, Sd = 0.593). In waterfall-type layout, users can browse all the information by simply sliding up and down the interface to quickly determine the knowledge they are interested in, while in juxtaposition-type layout, there is an additional gesture of sliding left and right, which invisibly affects the operation efficiency. A similar conclusion has been drawn from the research on interface design in the field of e-commerce, i.e., the vertical layout is more attractive to consumers than the horizontal layout [48]. The conclusion also indicates that waterfall-type interface layout is more in line with the information search strategy of young people, because the elderly are exclusively used to the interface layout that scrolls up and down [17,47]. Therefore, we have reason to believe that when the subjects are not college students but the elderly, the results of the study are likely to change. Yi et al. [49] reported that educational level affected the user’s preference for interface layout, and the user group with a higher educational level (such as college students) preferred an interface with rich operational gestures, which was contrary to our finding. In our study, compared to the waterfall-type layout, juxtaposition-type layout adds the gesture of sliding left and right but it does not receive preference because of this new gesture. Some participants stated that waterfall-type layout allowed them to browse information “without thinking”, while in juxtaposition-type layout, they needed to select a topic of interest before reading. Therefore, Hypothesis 2 is rejected because there are significant differences in system usability between the different interface layouts.

The third research question is: what is the interaction between interface emotion and interface layout? To the best of our knowledge, previous studies did not discuss the interaction between interface emotion and interface layout in the context of digital public health safety education, so no existing research results can be compared with our conclusions. It is found that interface emotion and interface layout are interactive, both in terms of “effort of the lesson” and “interface quality”, with similar results. We speculate that the reason for this conclusion is that working efficiency and learning ability makes people feel optimistic when selecting a target and plan [12], while waterfall-type layout is less complicated. Hence, a positive emotional interface with waterfall-type layout requires participants to make less effort and is believed to show good interface quality. In contrast, a negative emotional interface with juxtaposition-type layout is preferred by participants.

Negative emotion is not conducive to the cognition process of readers, so that they may need to take longer time to understand the text content [11,42]. However, juxtaposition-type layout may bring a certain “sense of order” and thus is beneficial in pacifying readers’ mood and balancing the anxiety caused by negative emotion. Hence, in negative emotional interface with juxtaposition-type layout, readers often subjectively feel that they make less effort. In the interview, some participants mentioned that juxtaposition-type layout contained more operational fun. For instance, sliding left and right or up and down contributes to relieving a sense of tension in negative emotion. Another important reason may be that, in juxtaposition-type layout, a learning task needs to be completed more by manual operation so that content with negative emotion read by participants is less than that read in waterfall-type layout at the same time and participants’ “information overload” in terms of negative emotional content is avoided. However, in waterfall-type layout, content with negative emotion read by participants is more than that read in juxtaposition-type layout at the same time, due to the operational convenience of this type of layout, so that participants experience a stronger sense of oppression. Therefore, Hypothesis 3 is rejected because there is a significant interaction between the interface emotion and the interface layout in course evaluation and system usability.

## 7. Conclusions

The impact of emotion on learning effectiveness is a long-term concern in the academic field. Positive emotion may distract students’ thinking, while negative emotion may facilitate concentration of attention [6,45], which depends on many factors such as the specific learning content, form and the mentality of the learner. In this research, by analyzing the impact of different interface design on readers’ understanding, course evaluation and system usability when receiving public health safety education, the following conclusions are obtained: first, different interface emotion can cause significant difference in the understanding of participants, where negative emotional interface has the best learning transfer effect; second, different interface emotion can lead to significant difference in the course evaluation score given by participants, where positive emotional interface has obvious advantages in terms of the “appeal of the lesson”, “enjoyment of the lesson” and “interface quality”; third, different interface layout will cause significantly different “system usability”, where waterfall-type layout is highly appraised by users; fourth, interface emotion and interface layout have similar interactive effect in terms of “effort of the lesson” and “interface quality”, where positive emotional interface with waterfall-type layout and negative emotional interface with juxtaposition-type layout are favored by users.

Like most researches, this research has some limitations. First of all, all participants were Chinese residents and the experiment was carried out in a Chinese context. Whether the research conclusion is applicable to other cultural contexts or not remains to be confirmed. Second, the number of participants might limit the ability to conduct extensive confirmation and expand the research findings. In addition, individual differences such as learners’ previous knowledge reserve, cognitive style, and ability will affect their emotions and learning outcomes after receiving external stimuli [42], which may affect the experimental results. It is necessary to conduct in-depth research on individual differences in the future. Third, some scholars presumed the existence of an arousal dimension (low or high; also called activating or deactivating) that may facilitate or impede the learning process [26,42,52]. Hence, future studies, taking into account the arousal dimension, may have more interesting findings. Fourth, due to time and labor constraints, there were some factors that were not covered by our investigation (such as whether the subjects’ families were infected with the virus, and the severity), which might be helpful for further interpretation of the experimental data. Finally, a quiet experimental site was selected to keep unnecessary factors under control. Therefore, the applicability of the conclusion in a more complicated scenario (i.e., in a scene with noise or learning while walking) is to be further verified. This research is expected to stimulate constructive debate on digital public health safety education.

## Figures and Tables

**Table 1:** Emotion recognition test results for the 6 interface designs.

Interface Design	1	2	3	4	5	6	7	8	9	10	11	Recognition Rate
A	6	3	4	2								100%
B					4	8	3					100%
C						1	1	3	5	5		86.7%
D	5	4	6									100%
E					3	6	6					100%
F						2		3	7	2	1	80%

A = positive emotional interface with waterfall-type layout, B = negative emotional interface with waterfall-type layout, C = neutral emotional interface with waterfall-type layout, D = positive emotional interface with juxtaposition-type layout, E = negative emotional interface with juxtaposition-type layout, F = neutral emotional interface with juxtaposition-type layout; positive vocabulary (1—amused; 2—delighted; 3—happy; 4—fun), negative vocabulary (5—scared; 6—serious; 7—strained), neutral vocabulary (8—placid; 9—serene; 10—calm), 11—none.

**Table 2:** Descriptive statistics and two-way ANOVA of the retention test.

Retention Test	Waterfall Type		Juxtaposition Type		M		Emotion	Layout	Emotion × Layout
	M	SD	M	SD	M	SD	*p*	*p*	*p*
A	6.50	2.141	5.86	1.491	6.18	1.864	0.298	0.506	0.258
B	6.38	1.561	6.64	2.022	6.51	1.800			
C	6.62	1.529	6.55	1.915	6.58	1.723			
M	6.50	1.756	6.35	1.843					

A = positive, B = negative, C = neutral.

**Table 3:** Descriptive statistics and two-way ANOVA of the transfer test.

Transfer Test	Waterfall Type		Juxtaposition Type		M		Emotion	Layout	Emotion × Layout
	M	SD	M	SD	M	SD	*p*	*p*	*p*
A	19.07	3.165	19.02	2.718	19.05	2.933	0.000	0.066	0.379
B	20.43	2.838	21.52	2.813	20.98	2.862			
C	17.81	4.538	19.02	2.975	18.42	3.863			
M	19.10	3.720	19.86	3.053					

A = positive, B = negative, C = neutral.

**Table 4:** Descriptive statistics and two-way ANOVA for the difficulty of the lesson.

Difficulty	Waterfall Type		Juxtaposition Type		M		Emotion	Layout	Emotion × Layout
	M	SD	M	SD	M	SD	*p*	*p*	*p*
A	2.31	0.924	1.83	0.794	2.07	0.889	0.201	0.147	0.074
B	2.26	0.912	2.07	0.997	2.17	0.955			
C	2.24	1.078	2.40	0.701	2.32	0.907			
M	2.27	0.967	2.10	0.866					

A = positive, B = negative, C = neutral.

**Table 5:** Descriptive statistics and two-way ANOVA for effort in the lesson.

Effort	Waterfall Type		Juxtaposition Type		M		Emotion	Layout	Emotion × Layout
	M	SD	M	SD	M	SD	*p*	*p*	*p*
A	2.55	0.772	3.24	0.850	2.89	0.878	0.063	0.415	0.000
B	3.52	0.943	2.81	0.707	3.17	0.903			
C	3.02	0.975	2.79	0.813	2.90	0.900			
M	3.03	0.979	2.94	0.813					

A = positive, B = negative, C = neutral.

**Table 6:** Descriptive statistics and two-way ANOVA for the appeal of the lesson.

Appeal	Waterfall Type		Juxtaposition Type		M		Emotion	Layout	Emotion × Layout
	M	SD	M	SD	M	SD	*p*	*p*	*p*
A	1.83	0.824	1.69	0.749	1.76	0.786	0.000	0.300	0.079
B	2.07	0.867	1.71	0.673	1.89	0.792			
C	2.29	0.805	2.48	0.804	2.38	0.805			
M	2.06	0.846	1.96	0.824					

A = positive, B = negative, C = neutral.

**Table 7:** Descriptive statistics and two-way ANOVA for desire for similar lessons.

Desire	Waterfall Type		Juxtaposition Type		M		Emotion	Layout	Emotion × Layout
	M	SD	M	SD	M	SD	*p*	*p*	*p*
A	2.33	0.902	2.21	0.813	2.27	0.855	0.781	0.596	0.896
B	2.29	0.774	2.24	0.821	2.26	0.793			
C	2.19	0.862	2.19	0.804	2.19	0.828			
M	2.27	0.843	2.21	0.806					

A = positive, B = negative, C = neutral.

**Table 8:** Descriptive statistics and two-way ANOVA for the enjoyment of the lesson.

Enjoyment	Waterfall Type		Juxtaposition Type		M		Emotion	Layout	Emotion × Layout
	M	SD	M	SD	M	SD	*p*	*p*	*p*
A	1.95	0.623	1.74	0.734	1.85	0.685	0.000	0.478	0.131
B	2.33	0.846	2.17	0.621	2.25	0.742			
C	2.19	0.707	2.38	0.697	2.29	0.704			
M	2.16	0.742	2.10	0.731					

A = positive, B = negative, C = neutral.

**Table 9:** Descriptive statistics and two-way ANOVA for system usability.

System Usability	Waterfall Type		Juxtaposition Type		M		Emotion	Layout	Emotion × Layout
	M	SD	M	SD	M	SD	*p*	*p*	*p*
A	2.08	0.445	2.77	0.663	2.43	0.662	0.180	0.000	0.121
B	2.34	0.723	2.77	0.491	2.56	0.652			
C	2.43	0.657	2.75	0.624	2.59	0.656			
M	2.28	0.633	2.77	0.593					

A = positive, B = negative, C = neutral.

**Table 10:** Descriptive statistics and two-way ANOVA for interface quality.

Interface Quality	Waterfall Type		Juxtaposition Type		M		Emotion	Layout	Emotion × Layout
	M	SD	M	SD	M	SD	*p*	*p*	*p*
A	1.86	0.451	2.17	0.518	2.01	0.508	0.000	0.166	0.000
B	2.78	0.564	2.07	0.565	2.43	0.666			
C	2.05	0.537	2.18	0.465	2.11	0.503			
M	2.23	0.652	2.14	0.516					

A = positive, B = negative, C = neutral.

## Data Availability

The data are not publicly available due to the data also forms part of an on-going study.
